# Navigating complexity: review and updates of endometriosis and adenomyosis imaging

**DOI:** 10.1093/bjr/tqag007

**Published:** 2026-01-08

**Authors:** Harmanjit Dev, Nathalie Marie Falkner, Emmeline Lee

**Affiliations:** Department of Radiology, Sir Charles Gairdner Hospital (SCGH), Nedlands, WA 6009, Australia; Department of Radiology, Sir Charles Gairdner Hospital (SCGH), Nedlands, WA 6009, Australia; Department of Radiology, Perth Children’s Hospital (PCH), Nedlands, WA 6009, Australia; Radiology, BreastScreen WA, Perth, WA 6000, Australia; Department of Radiology, Sir Charles Gairdner Hospital (SCGH), Nedlands, WA 6009, Australia; School of Medicine, University of Western Australia, Crawley, WA 6009, Australia; Department of Radiology, King Edward Memorial Hospital (KEMH), Subiaco, WA 6008, Australia; Western Ultrasound for Women, West Leederville, WA 6007, Australia

**Keywords:** endometriosis, adenomyosis, pelvic pain, infertility, radiology

## Abstract

Endometriosis and adenomyosis are prevalent chronic inflammatory conditions that significantly impact reproductive-age women, leading to debilitating symptoms such as pelvic pain, dysmenorrhea and infertility. Endometriosis is characterized by ectopic endometrial tissue outside the uterus, while adenomyosis involves endometrial deposits within the myometrium, often causing overlapping symptoms. Accurate diagnosis is essential for effective management, yet challenges persist due to variability in lesion location and presentation. Imaging plays a crucial role in the diagnosis, staging, and management of these conditions. While transvaginal ultrasound (TVUS) remains a reliable first-line tool with high specificity, magnetic resonance imaging (MRI) offers superior sensitivity, particularly for endometriomas and deep endometriosis. Both modalities have their limitations but are complementary in preoperative assessment and treatment planning. Emerging imaging protocols, classification systems, and advancements such as dynamic TVUS and improved MRI techniques are discussed. Despite significant progress, the lack of a universally accepted classification system and standardized imaging protocols highlights the need for further research. Advancing diagnostic accuracy and treatment strategies through imaging holds promise for improving patient outcomes. This article reviews the pathophysiology, staging and subtypes of endometriosis and adenomyosis. Recent society guidelines are addressed and readers are provided with updates on imaging technologies with insights into future directions.

## Introduction

Endometriosis is a common chronic inflammatory disease affecting up to 10% of reproductive age women worldwide, associated with pelvic pain, dysmenorrhea, and infertility.^1^^,^^2^ Although historically deemed a gynaecological condition, it is more accurately considered a systemic disease.^1^ It is characterized by endometrium-like tissue outside the uterus, while adenomyosis features endometrial deposits within the myometrium associated with smooth muscle hypertrophy. Endometriosis causes pelvic pain, dysmenorrhoea, dyspareunia, menorrhagia, infertility, bowel and urinary complications and is linked to fatigue, anxiety and depression. Symptoms overlap with adenomyosis, which is most often associated with dysmenorrhoea, menorrhagia and subfertility.^3^^,^^4^

Endometriosis is most commonly, but not exclusively, a disease of women of reproductive age, with an estimated prevalence of 5%-10%.^2^ It affects up to 50%-80% of reproductive-age women with chronic pelvic pain and 50% of those with infertility.^1^^,^^2^ Delays to diagnosis and treatment of endometriosis are the norm, commonly ranging from 4 to 11 years from symptom onset.^1^ Endometriosis affects adolescents and postmenopausal women, presenting unique challenges in these groups. It is the leading cause of dysmenorrhea and chronic pelvic pain in adolescents, occurring in up to 38% of cases, most frequently as superficial peritoneal disease.^5^^,^^6^ There is an estimated endometriosis prevalence of up to 5% in post-menopausal women, usually affecting the ovary.^7^

The exact prevalence of adenomyosis is unclear, but imaging studies suggest it is more common in reproductive-age women than previously thought, with rates of 20%-35% and may occur in nulliparous or parous women.^8–10^ Adenomyosis has been reported in 25% to 70% of patients with endometriosis, with a prevalence of 49% to 66% specifically observed in those with deep endometriosis (DE).^11^

Medical imaging plays a critical role in diagnosis, treatment planning and monitoring for complications of both endometriosis and adenomyosis.

## Pathophysiology

Endometriosis and adenomyosis share common pathophysiological pathways.^12^ Theories on endometriosis pathogenesis are varied, with no single theory fully explaining all cases. The disease is likely multifactorial, involving hormonal, environmental, genetic and immunological factors.^1^^,^^13–15^ The retrograde menstruation theory, the most widely accepted, suggests ectopic deposition of endometrial tissue through retrograde flow. This is supported by the dependent distribution of deposits and its link to obstructive congenital Müllerian duct anomalies.^3^ Additionally, the increased risk and severity of endometriosis in individuals with an affected first-degree relative, along with high concordance in twin studies, suggest a genetic link.^1^ However, the relative contributions and mechanisms of identified genetic variants in pathogenesis remains unclear. An environment of oestrogen excess and intrinsic progesterone resistance support endometriotic implant growth, with amplification of acute and chronic inflammation caused by inflammatory cytokines, chemokines and prostaglandins.^1^

In adenomyosis, the presence of endometriotic tissue within the myometrium is believed to arise from repeated injury to the endometrial-myometrial junction, often caused by menstruation and pregnancy. This may result in endometrial tissue invagination or metaplastic transformation of progenitor cells.^16^

## Phenotypes of endometriosis

Endometriosis is classified into 3 main types: superficial, deep and ovarian endometriomas. Extra-pelvic endometriosis, on the other hand, is a rare entity which occurs relatively infrequently compared to pelvic disease.

Superficial lesions, found in 15%-80% of cases, typically occur on the pelvic peritoneal surface, with variable appearances (red, black, white, or clear) and may be symptomatic or incidental.^3^^,^^17–19^ These lesions do not show subperitoneal extension histologically.^20^

Deep Endometriosis (DE) refers to endometriotic deposits in the abdomen or pelvis extending to any depth beneath the peritoneal surface, often invading the muscularis propria of hollow viscera. As the most severe form of endometriosis, DE is typically nodular and fibrotic, causing tissue distortion. Previously termed deeply infiltrating endometriosis, it was defined by lesions infiltrating more than 5 mm below the peritoneum, but this depth criterion was recently removed due to measurement inaccuracies.^18^^,^^19^^,^^21–23^ It is seen in up to 20% of cases and is most frequently found in the pelvis, involving fibromuscular structures including the uterine ligaments, vagina, rectum and bladder.^24^ With respect to the bowel, DE invasion of the muscularis is considered severe disease. The surgical management of DE varies significantly according to location and extent, potentially necessitating a combined approach with input from Gynaecologic, Colorectal and Urology Surgical teams.

**Figure 1.**
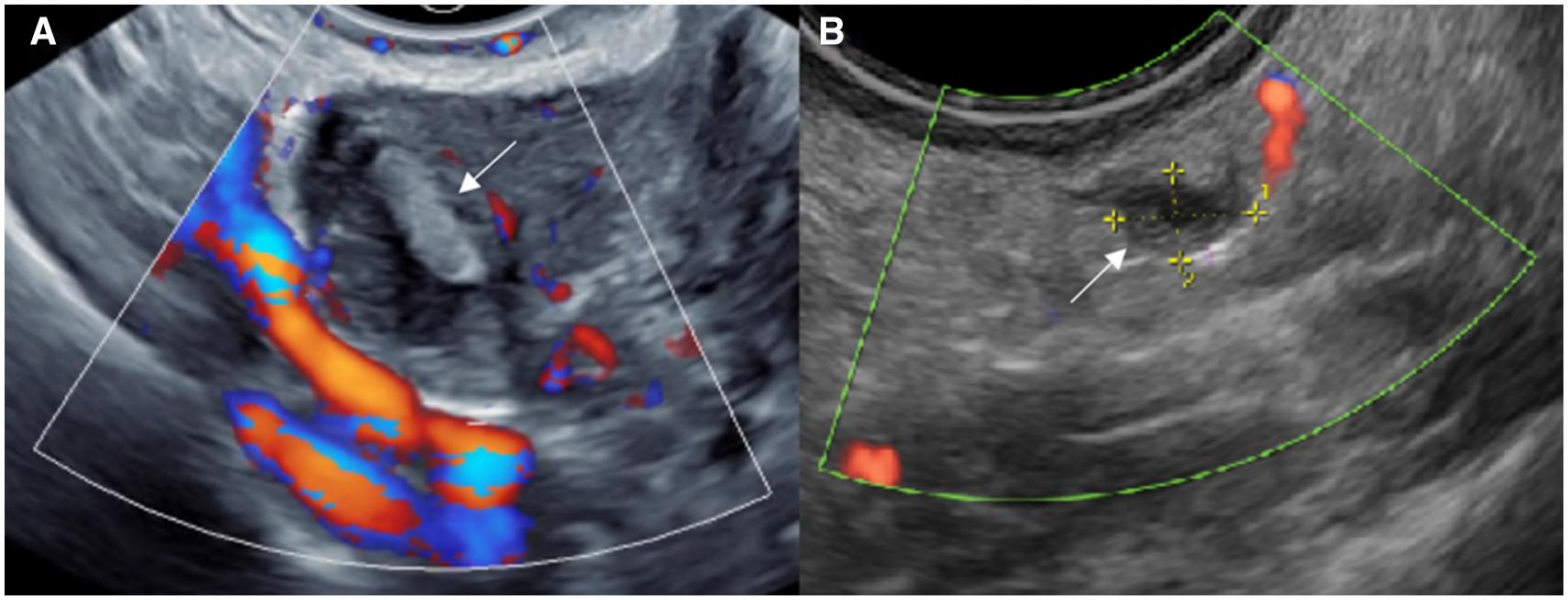
A 44-year-old female underwent a pelvic ultrasound for investigation of heavy menstrual bleeding. Transvaginal ultrasound revealed multiple bilateral endometriomas with fluid-fluid levels consistent with bleeding occurring at different time points and right uterosacral DE. (A) Right ovarian endometrioma containing avascular echogenic internal material (arrow), in keeping with blood products. (B) Right uterosacral ligament endometriotic deposit (arrow), consistent with DE.

Ovarian endometriotic cyst or endometrioma may result from invagination or true cyst formation, with the wall containing endometrium-like tissue. The cysts usually contain blood-stained dark syrup-like fluid, with the colour and consistency giving rise to the name “chocolate cysts” (Figures 1 and 5).^19^ Endometriomas have been reported to occur in up to 44% of patients with endometriosis, with bilateral ovarian involvement reported in 1/3 to 1/2 of cases (Figure 1).^3^

Extrapelvic endometriosis refers to endometrium-like tissue outside the abdominal cavity (including, but not limited to, the thorax, nervous system and skeletal muscle).^19^^,^^25^ Iatrogenic endometriosis, defined as lesions resulting from direct or indirect dissemination of endometrium following or during surgery, is considered as a distinct subtype, most commonly seen in the abdominal wall.

## Distribution and complications

### Deep pelvic endometriosis

The uterosacral ligaments (USL), extending from the torus uterinus to the sacrum on each side, are involved in 60%-85% of endometriosis patients at laparoscopy, making them the most common site of DE.^20^ Other commonly involved pelvic fibromuscular sites of DE include the torus uterinus, parametrium and rectovaginal space. Severe DE at the USL or torus uterinus can extend to involve the vagina, the rectouterine space and rectovaginal septum, the rectosigmoid colon, ureters and pelvic nerves.^20^

### Intestinal endometriosis

The bowel is a common site of extragenital endometriosis, seen in up to 37% of patients with DE. The rectum and sigmoid colon are the most commonly involved sites (accounting for 70%-93% of intestinal endometriosis), often contiguous with retrocervical endometriosis.^20^^,^^26^ The effect of blood pooling in the pouch of Douglas is theorized to explain this locational predisposition.^27^ Less frequently, it involves the ileum, appendix and caecum. DE often infiltrates the bowel wall, causing fibrosis, adhesions and severe pain due to neurogenesis in bowel nodules.^27^^,^^28^ Therefore, bowel endometriosis should be considered in patients with symptoms such as dyspareunia and dysmenorrhea. TVUS is recommended as the first-line imaging modality, enabling evaluation for deposits, quantification of size, depth and associated luminal stenosis.^28^

### Urinary tract endometriosis

**Figure 2.**
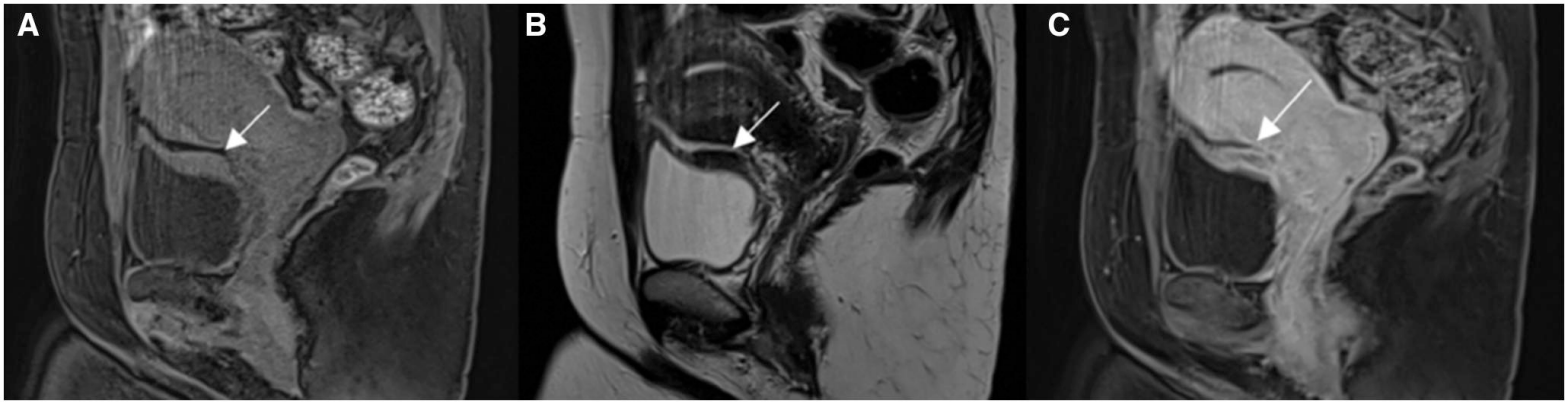
47-year-old female with biopsy-proven bladder wall endometriosis (arrow). MRI pelvis demonstrates thickening of the bladder dome with (A) intermediate signal on pre-contrast fat saturated T1weighted imaging, (B) low signal on T2 weighted imaging and (C) inhomogeneous enhancement post-contrast administration.

Urinary tract endometriosis (UTE) is rare, affecting 0.3%-12% of endometriosis patients.^29^ The mechanism of UTE remains unclear and may be classified as primary (spontaneous) or secondary (following iatrogenic injury). Most commonly associated with the DE subtype, it carries a risk of developing obstructive uropathy. The bladder is affected in up to 85% of urinary tract endometriosis cases, with deposits typically in the detrusor muscle and epithelium (Figure 2).^30^^,^^31^ Patients with bladder endometriosis may report urinary symptoms like suprapubic pain, dysuria and haematuria. Combining clinical examination with ultrasound has shown to increase sensitivity in detecting bladder endometriosis and as such, assessment of the urinary tract is recommended in those undergoing imaging for endometriosis.^30^

### Abdominal wall endometriosis

**Figure 3.**
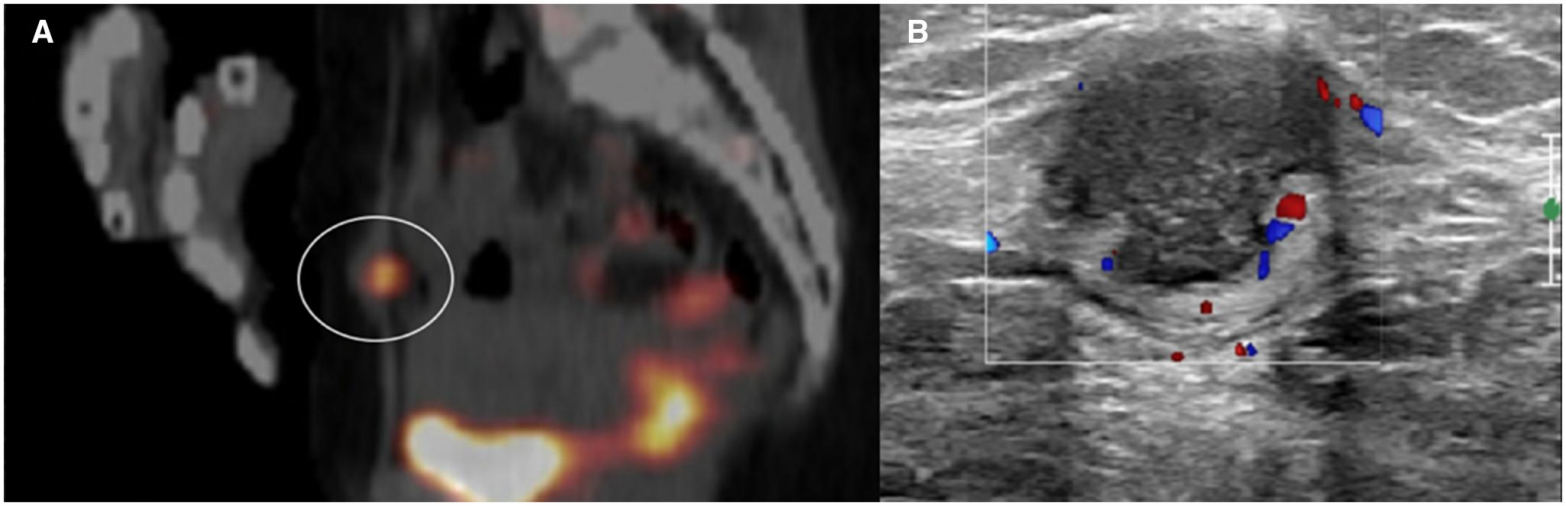
34-year-old female undergoing FDG PET/CT (A) for papillary thyroid carcinoma, which identified an FDG-avid subcutaneous anterior abdominal wall nodule (circled). (B) Further evaluation with US showed a non-tender, non-compressible lower abdominal wall nodule with irregular, hyperechoic margins and homogenous internal echoes with posterior acoustic enhancement. Mild peripheral vascularity is noted. This nodule was proven to be benign endometriosis on ultrasound-guided fine needle aspiration.

Abdominal wall endometriosis (AWE) is an uncommon form of endometriosis, often linked to prior abdominal surgery (Figure 3). Patients typically present with cyclical abdominal pain and a palpable mass. While diagnosis requires tissue confirmation, pre-operative imaging aids in differentiating AWE from other soft tissue lesions.^32^ Ultrasound is the first-line imaging modality for diagnosis; however, MRI is recommended to evaluate nodules larger than 3 cm to assist in surgical planning.^32^ These lesions are generally isoechoic or hypoechoic on US with internal or peripheral vascularity.^33^

### Endometriosis in pregnancy

In pregnancy, endometriomas may develop enlarging vascularized solid tissue due to decidualization, particularly during the first two trimesters. These changes may mimic malignancy and when identified require serial imaging and correlation with CA-125.^34^ Some cases of DE and endometriomas may regress during pregnancy, whereas others may persist or grow but typically reduce in size after childbirth.^22^

Endometriosis may also result in adverse pregnancy and obstetric outcomes due to multiple pathophysiological mechanisms, including activation of abnormal inflammatory pathways. Other causes include modifications in the structural and functional zones of the uterus and altered uterine peristalsis which can then increase the risk of placenta praevia.^35^ A recent retrospective study evaluating over 3800 patients reported a higher incidence of placenta praevia and volume of postpartum haemorrhage in endometriosis patients.^36^ Other common obstetric complications associated with endometriosis and adenomyosis include preterm delivery, pre-eclampsia and haemorrhage.^35^^,^^37^^,^^38^ Acute pregnancy complications from adenomyosis, like haemoperitoneum and visceral perforation are rare but may be life-threatening.^38^

### Malignant transformation

Although endometriosis is a benign condition, several studies show an associated risk of malignant transformation and highlight its association with various cancers, particularly epithelial ovarian cancers.^39–41^ Malignant transformation is thought to occur in 1% of patients with endometriosis and is ovarian in 75% of cases.^20^^,^^42^ Barnard et al^39^ showed that women with endometriosis had a 4.2-fold higher risk of developing ovarian cancer than those without endometriosis. The ectopic endometrium-like tissue is thought to serve as the tissue of origin for both endometrioid and clear cell ovarian cancers.^39^ Oestrogen stimulation, chronic inflammation and inactivation of tumour suppressor genes likely contribute to the generation of a microenvironment prone to genetic alterations leading to malignant transformation.^39^^,^^43^ Endometriosis has also been associated with an increased risk of developing other cancers including thyroid and breast cancer.^44^

## Classification and staging

### Endometriosis

Historical endometriosis classification systems show variable reproducibility, validation and correlation with symptoms or treatment outcomes, resulting in a lack of international consensus. The widely used revised American Society of Reproductive Medicine (rASRM) classification stages disease based on laparoscopy but poorly correlates with pain and surgical complexity (Table 1). The American Association of Gynaecologic Laparoscopists (AAGL) Special Interest Group in Endometriosis created the 2021 Endometriosis Classification System, which offers an anatomy-based surgical scoring system that more robustly predicts surgical complexity than rASRM.^45^

**Table 1.** Summary of the rASRM classification of endometriosis staging.^4^^,^^84^

Stage	Points	Description
I	1-5	Minimal disease with few superficial deposits.
II	6-15	Mild disease with a greater number and depth of deposits.
III	16-40	Moderate disease with many deep deposits, adhesions and small ovarian cysts.
IV	>40	Severe disease with multiple deep deposits, large ovarian cysts and dense adhesions.

**Figure 4.**
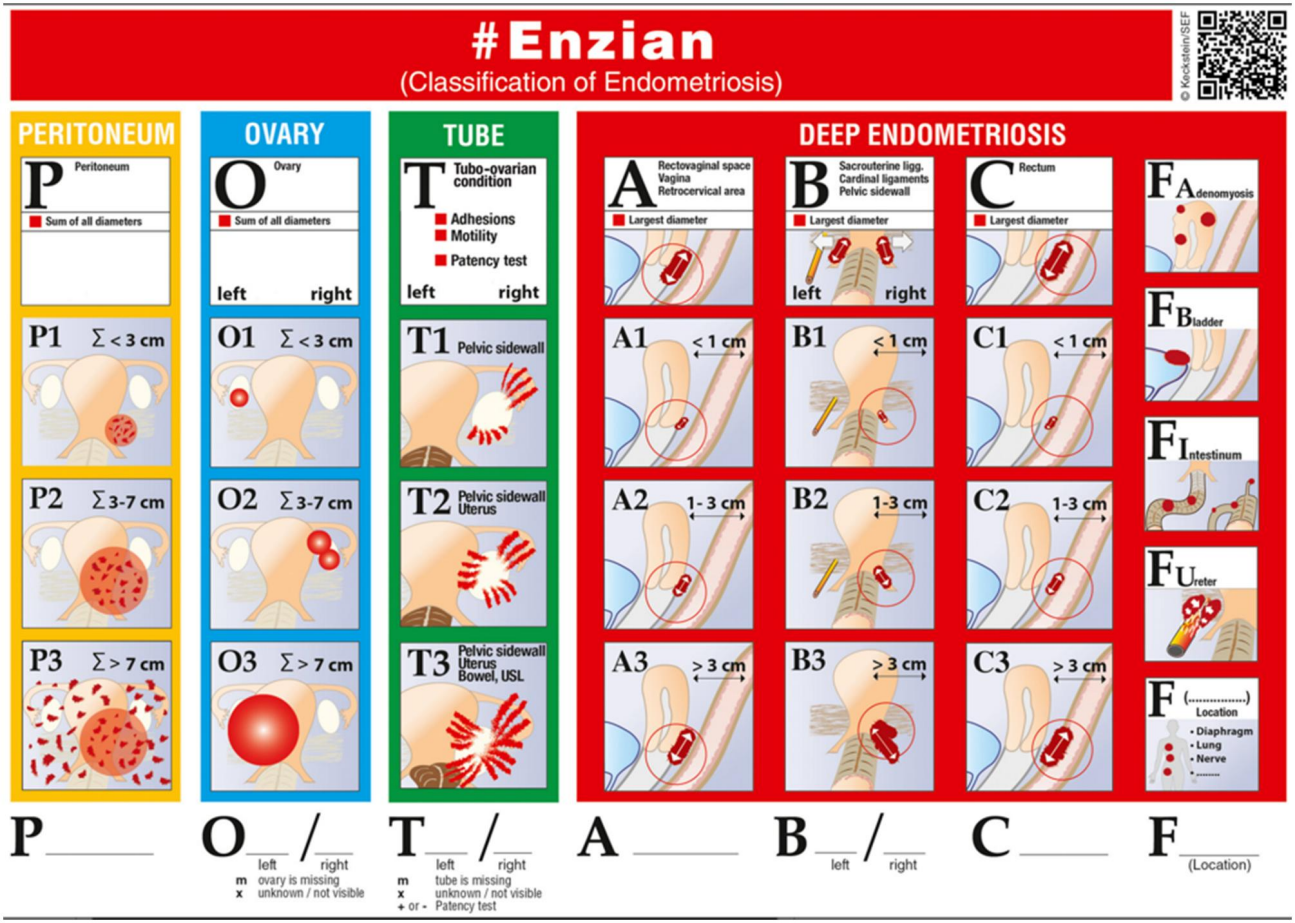
#Enzian classification overview with potentially affected organs and compartments, reproduced with permission from Keckstein et al.^46^ This classification builds on the previous Enzian classification which categorizes DE lesions into 3 compartments: A (vagina, rectovaginal space and retrocervical area), B (uterosacral ligaments, cardinal ligaments, pelvic sidewall) and C (rectum). It also includes “F” locations, such as adenomyosis (FA), the bladder (FB), intestine (FI), ureters (FU) and other extragenital sites (F…). The more recently developed #Enzian system includes sums of deposits on the peritoneum (P), sum of ovarian endometriomas (O) and fallopian tube/tubo-ovarian complex endometriosis (T), assessing for adhesions, mobility and patency.^4^

The recently developed #Enzian classification builds upon the original DE-focused Enzian system to address the burden of endometriosis more comprehensively (Figure 4). It includes ovarian, tubal and peritoneal endometriosis, adenomyosis and extra-abdominal disease. A key advantage is its integration of imaging-based classification with surgical staging, accounting for the strengths and limitations of both approaches.^4^^,^^46^

### Adenomyosis

Multiple classification systems for adenomyosis have been proposed based on both histopathology or imaging bases, but, similar to endometriosis, there is no universally accepted classification system and existing systems require ongoing validation.^47^

In 2018, Lazzeri et al proposed an ultrasound-based classification system, defining adenomyosis as diffuse, focal or adenomyoma with further subdivision according to sonographic characteristics. This system has demonstrated reproducibility and clinical relevance, but requires further validation.^8^^,^^48^ Further TVUS classification systems have been proposed incorporating MUSA criteria, which include lesion location, type, size, extent of uterine involvement and presence of cysts etc, though these also require external validation. ^48^

In the 2022 Delphi revision of the MUSA criteria, the ultrasound signs of adenomyosis were stratified into direct and indirect categories, with the presence of at least one direct feature required to support diagnosis. Direct (typical) features of adenomyosis include the presence of myometrial cysts, hyperechoic islands and echogenic subendometrial lines or buds. Indirect features are secondary to ectopic endometrium in the myometrium and include a globular uterus, asymmetrical myometrial thickening, fan-shaped shadowing, translesional vascularity, and an irregular or interrupted junctional zone.^49^^,^^50^

Additionally, Kobayashi and Matsubara issued one of the most comprehensive MRI based classification systems for adenomyosis, which evaluates 5 parameters: the affected area (internal or external myometrium), pattern (diffuse or focal), size, location (anterior, posterior, lateral, or fundal), and presence of associated conditions (such as endometriosis and fibroids).^48^^,^^51^

## Imaging

In 2024, the International Society of Ultrasound in Obstetrics and Gynaecology (ISUOG) released an International Consensus Statement outlining evidence-based and clinically relevant recommendations for the use of non-invasive imaging techniques to diagnose and classify pelvic DE (Table 2).^52^ Whilst laparoscopy remains the gold standard for diagnosis, modern TVUS and MRI are valuable tools which complement laparoscopy and are recommended for pre-operative assessment. There is continued debate regarding the use of non-invasive classification systems in combination with TVUS or MRI, with the majority agreeing strongly to a combination of the #Enzian classification with TVUS or MRI, with recognized limitations.

**Table 2.** Summary of recommendations from the ISUOG International Consensus Statement on the use of non-invasive imaging techniques.

Category	Key recommendations	Evidence level	Consensus (%)
General statements	Imaging accuracy depends on operator skill. Pre-operative imaging by trained operators is recommended.	1a	96.2%
Transvaginal US (TVUS)	First-line imaging for endometriosis detection; effective for rectum, rectovaginal septum, vagina, bladder and uterosacral ligaments (USL).	1a	73.6%-90.6%
MRI	Reliable for rectosigmoid, rectovaginal septum, vagina, bladder, and USL; superior to CT for most cases.	1a	86.8%-92.5%
CT	Less studied; no clear advantage over MRI; limited use due to radiation exposure with insufficient evidence to support its use for detection of pelvic DE over other imaging modalities	2a	69.8%-90.6%

Adapted from Condous et al.^52^

### Transvaginal ultrasound

#### Endometriosis

**Figure 5.**
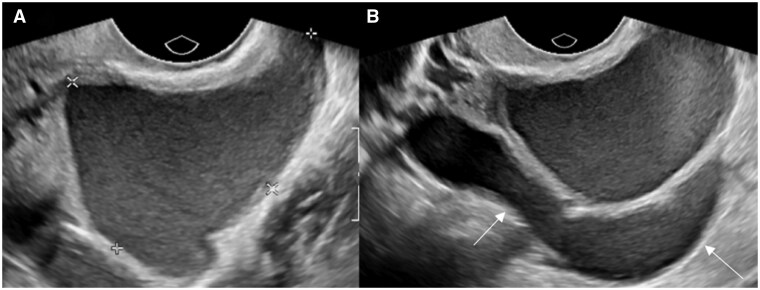
Transvaginal ultrasound of a 51-year-old female showing (A) a left ovarian unilocular cyst with ground glass internal echoes consistent with an endometrioma (with contour deformity related to probe pressure) (B) Haematosalpinx was also noted.

Transvaginal ultrasound is recommended as the first-line imaging modality for assessment of endometriosis due to its accessibility, low cost and ability to perform dynamic assessment. A high-frequency endocavitary transducer, typically up to 9 MHz and preferably with 3-dimensional (3D) capabilities for lateral plane steering, is suggested.^53^ The test performance of TVUS varies in the existing literature, with reported sensitivities and specificities of 57%-98% and 87%-100%, respectively, for DE and 93% and 96% for ovarian endometrioma.^23^ The diagnostic performance of ultrasound in the detection of DE varies significantly depending on the site affected, showing highest sensitivity in the detection of rectosigmoid DE (85%-94%) and relatively limited sensitivity in the detection of rectovaginal septum and uterosacral ligament DE (59% and 67%, respectively).^54^ The role of ultrasound in the identification of superficial endometriosis and fallopian tube involvement remains comparatively limited (Figure 5).

Accuracy and efficiency of diagnosis using TVUS is dependent on sonographer experience and technical skills. The International DE Analysis (IDEA) Consensus recommends a 4-step approach when evaluating endometriosis on US. These steps include assessing the uterus and adnexa, evaluating soft markers, examining the Pouch of Douglas using the sliding sign, and identifying deep endometriotic nodules in the pelvic compartments.^22^ The IDEA consensus was recently revised in 2025, where it highlighted the use of TVUS as a key first-line imaging tool for the evaluation of endometriosis, including superficial disease.^55^ Young et al^53^ summarize a compartment-based US protocol for sonographers for subsequent evaluation by radiologists (Table 3).

**Table 3.** Summarized US protocol for sonographer evaluation of DE as proposed by Young et al.^53^

Compartment	Key structures assessed	Approach
Anterior	Bladder and vesicouterine pouch	Examine bladder and vesicouterine pouch for nodules. Assess for adhesions using the transducer and observe sliding between the bladder and uterus.
Middle	Ovaries and adjacent anatomy	Assess ovarian mobility by applying pressure with the transducer or manual suprapubic pressure. Look for adhesions to the uterus, bowel, or contralateral ovary.
Posterior	Rectosigmoid colon, uterosacral ligaments and cul-de-sac	Use the sliding sign to evaluate uterine mobility relative to posterior structures.

**Figure 6.**
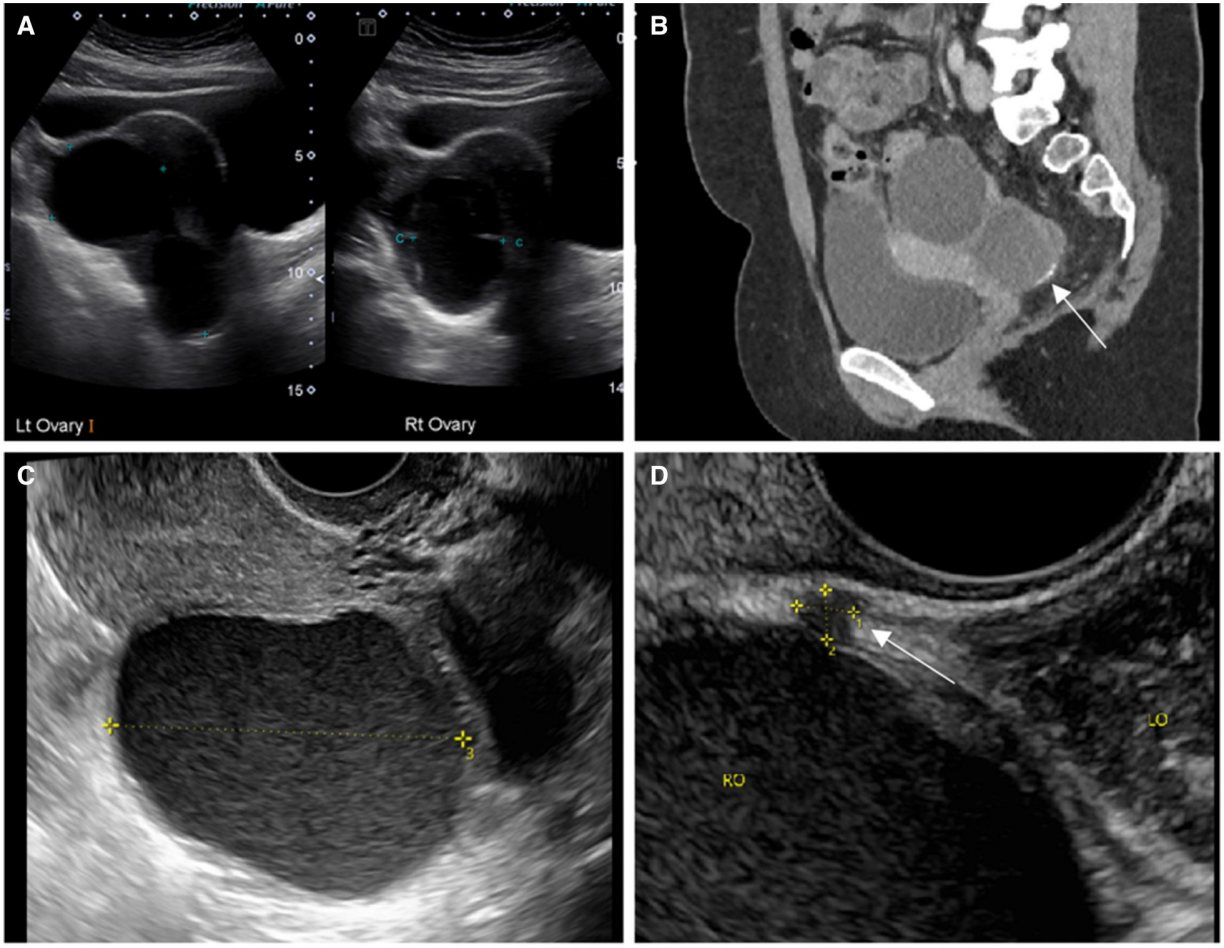
A 29-year-old female presented to the emergency department with sudden onset of right lower abdominal pain and nausea. (A) Transabdominal US showed enlarged ovaries with multiple cysts. (B) Computed tomography (CT) showed multiple cysts arising from both ovaries with rim calcification demonstrated in one of the left ovarian cysts (arrow). TVUS performed 14 days later showed multiple endometriomas, measuring up to 49mm, with internal echoes and fluid-fluid levels(C). A DE assessment (D) further demonstrated a 4mm right uterosacral ligament nodule (arrow).

Regarding ultrasound detection of ovarian endometriomas, the 2016 Cochrane review by Nisenblat et al reported sensitivity of 93% and specificity of 96%, with the study by Leonardi et al in 2022 showing similar ranges of 91%-92% for both sensitivity and specificity.^56–58^ The multicentre study by Montanari et al^59^ also reported that pre-operative non-invasive US assessment had a concordance rate with surgery ranging between 71% and 92%. Ovarian endometriomas on TVUS are measured in 3 orthogonal planes and usually appear unilocular or multilocular with ground-glass echogenicity and minimal vascularity (Figure 6). Concomitant DE of the pelvic wall is common. Atypical cases may show avascular papillary projections from fibrin strands.^21^^,^^56^ Endometriomas should be classified using the International Ovarian Tumour Analysis (IOTA) or O-RADS systems to enable differentiation from ovarian malignancies.^21^^,^^56^ Malignant endometriomas are more likely to be heterogeneous and multilocular, contain solid components or papillary projections with detectable colour Doppler flow.^22^

Several soft markers have been shown to be associated with the presence of superficial endometriosis, including ovarian mobility and site-specific tenderness.^54^ Whilst ovarian mobility shows variable diagnostic performance in the literature, ovarian immobility has been shown to be associated with both the presence of sidewall endometriosis/adhesions and the need for more complex surgery.^54^^,^^60^

**Figure 7.**
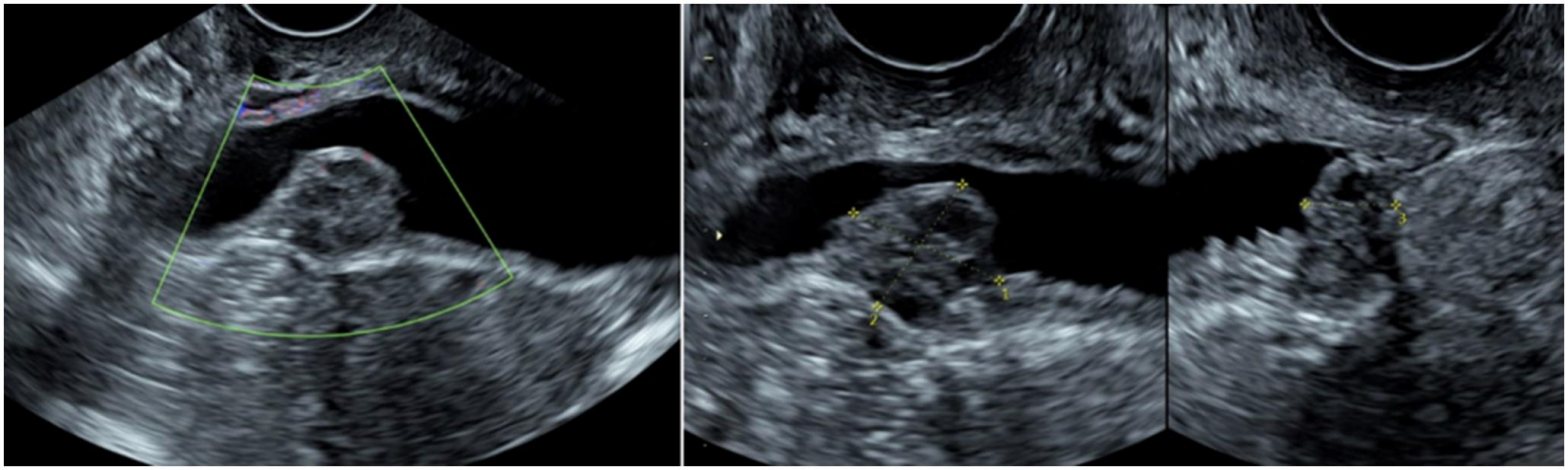
47-year-old female who presented with haematuria and a 10-year history of infertility. US shows a 13 × 12 × 7 mm heterogeneous and minimally vascular solid mass in the posterior bladder wall. This was later confirmed to be bladder endometriosis on histopathology.

Ultrasound shows variable diagnostic performance in the diagnosis of bladder DE, ranging from limited to adequate sensitivity (55%-71%) but robust specificity of 99%-100%.^54^ Bladder nodules appear as hypoechoic masses with irregular borders and possible internal cystic areas, penetrating at least the detrusor muscle (Figure 7). Transvaginal ultrasound with a full bladder enhances visualization, so bladder assessment is best performed later in the exam. A “negative sliding sign” between the bladder and uterus suggests adhesions in the vesicouterine pouch.^21^ Transabdominal US is an important component of the examination in the exclusion of hydronephrosis and is used for assessing extrapelvic endometriosis.^56^

**Figure 8.**
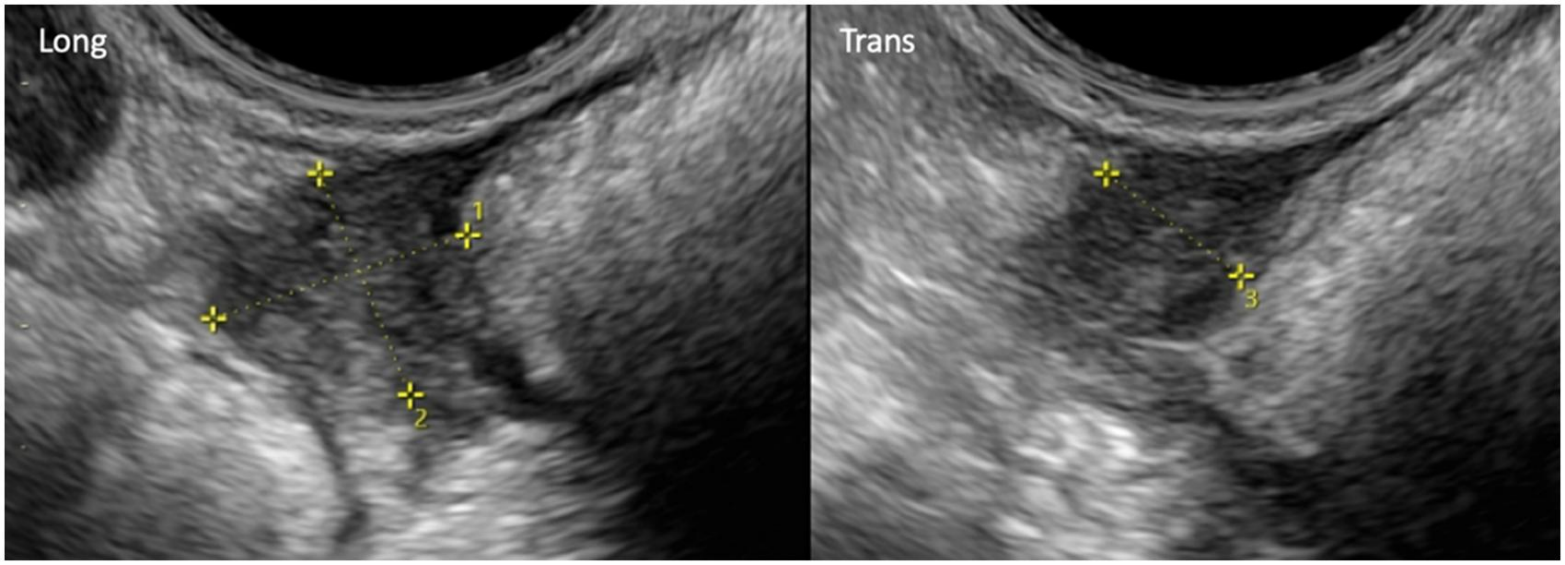
A 48-year-old female with a known small left ovarian endometrioma and a family history of ovarian and bowel cancer. Sonographic evaluation of the posterior compartment demonstrated a bowel nodule measuring 11 × 10 × 7 mm, likely involving the sigmoid colon, consistent with bowel deep endometriosis.

Bowel DE deposits appear as hypoechoic or isoechoic thickening or nodules with smooth or irregular margins which may infiltrate into the bowel wall (Figure 8).^54^ Deposits of DE should be measured in multiple planes and the distance from the anus should be recorded. For pre-operative planning, it is recommended to precisely identify lower anterior rectal lesions (situated below the level of the uterosacral ligaments) since these lesions can be especially challenging to excise.^21^ Accurate localization is crucial for optimizing surgical strategies and minimizing the risk of complications.^21^

Multiple enhanced TVUS techniques have been proposed, aiming to improve diagnostic accuracy, particularly in the assessment for DE in the posterior compartment.^54^ Dynamic assessment aids in detection of adhesions.^20^ Sonovaginography, involving the introduction of saline solution or US gel into the posterior vaginal fornix prior to inserting the US probe creates a dedicated acoustic window which can improve visibility and detection of DE without increased patient discomfort.^21^ Similarly, rectal water contrast US enables improved detection of rectosigmoid endometriosis and depth of luminal invasion through rectal luminal distension.^21^^,^^54^ Transrectal US has been shown to have similar accuracy to TVUS in the detection of posterior endometriosis but may be less well tolerated.^54^

Furthermore, sonoPODography is a recently described technique involving saline-infusion of the Pouch of Douglas via the Fallopian tubes, enabling the identification of superficial peritoneal lesions in contrast to the infused fluid. This technique has shown adequate sensitivity but excellent specificity of 100%.^61^

#### Adenomyosis

**Figure 9.**
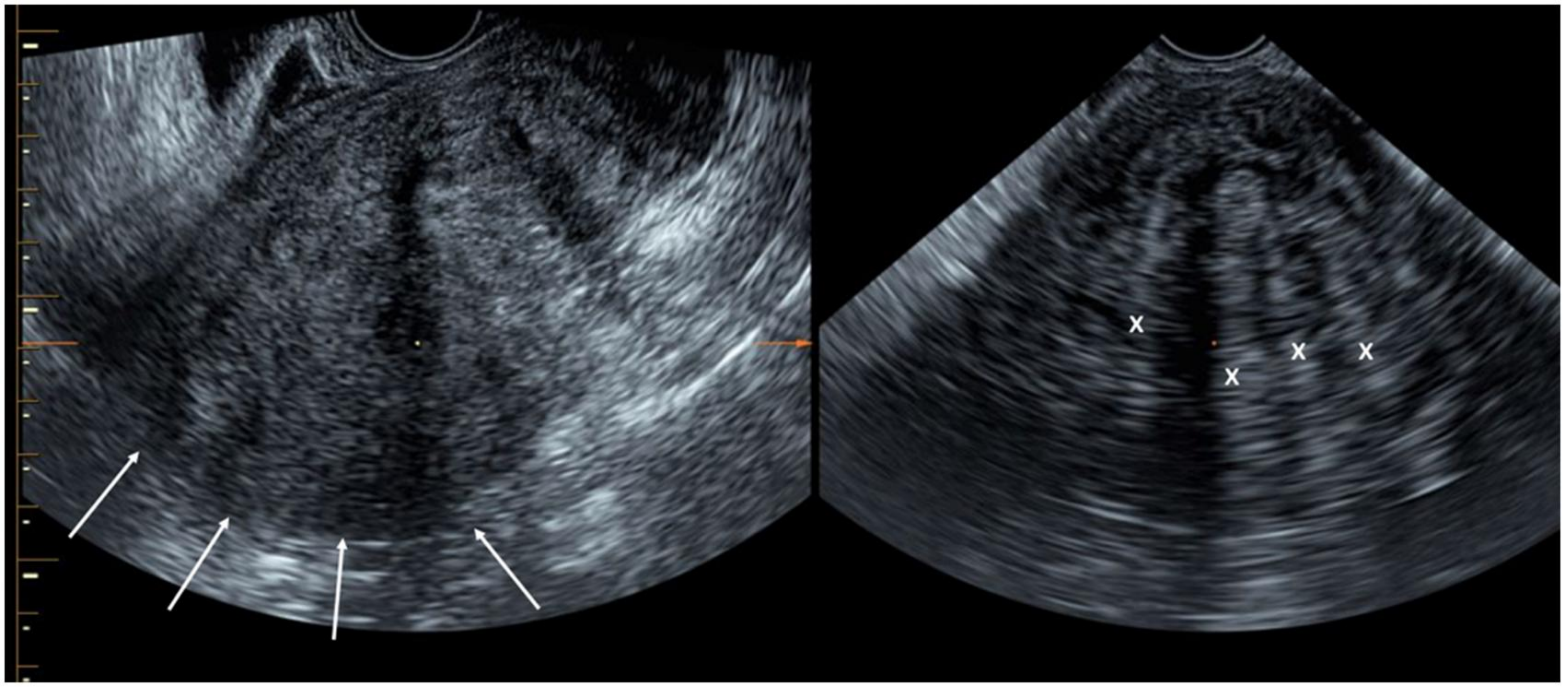
Long and transverse ultrasound images of a 43-year-old female with known Müllerian Duct anomaly (not characterised on these images) and endometriosis showing anteverted retroflexed uterus with a globular fundus (arrows), hyperechoic islands, heterogeneous myometrium with loss of clarity of the normal junctional zone and fan-shaped shadowing (x), which are suggestive of diffuse adenomyosis. It is important to note that the presence of hyperechoic islands is a direct ultrasound feature, while the remainder of the described sonographic findings represent the indirect features of adenomyosis as defined by the revised MUSA criteria.

Transvaginal ultrasound is a relatively cheap and accessible modality with proven effectiveness in the assessment of adenomyosis (Figure 9).^62^ The systematic review and head-to-head meta-analysis by Alcázar et al (2023) demonstrated similar diagnostic performance of TVUS and MRI in the detection of adenomyosis, with high specificity (81% for TVUS vs 80% for MRI) and moderate sensitivity (75% for TVUS vs 69% for MRI).^63^ Incorporating 3D imaging improves evaluation of the junctional zone, which enhances diagnostic precision. A combined approach using both 2D and 3D imaging is advised for optimal assessment.^64^ Hyperechoic endometrial foci within the myometrium are highly specific for adenomyosis and often coexist with hypoechoic areas, creating a heterogeneous echotexture. These changes, along with vertical stripes known as venetian blind shadowing, contribute to uterine enlargement and asymmetrical wall thickening.^64^

In 2023 Krentel et al. examined individual and combined ultrasound features for diagnosing adenomyosis.^65^ Subendometrial microcysts, myometrial cysts, and hyperechoic foci were identified as the most accurate markers on 2D TVUS, while a heterogeneous myometrium showed lower specificity due to overlap with other conditions. Combining features such as a bulky uterus, an ill-defined junctional zone and heterogeneous myometrium enhanced diagnostic accuracy, with the addition of subendometrial microcysts significantly reducing false-positive rates.^65^

### MRI

#### Endometriosis

**Figure 10.**
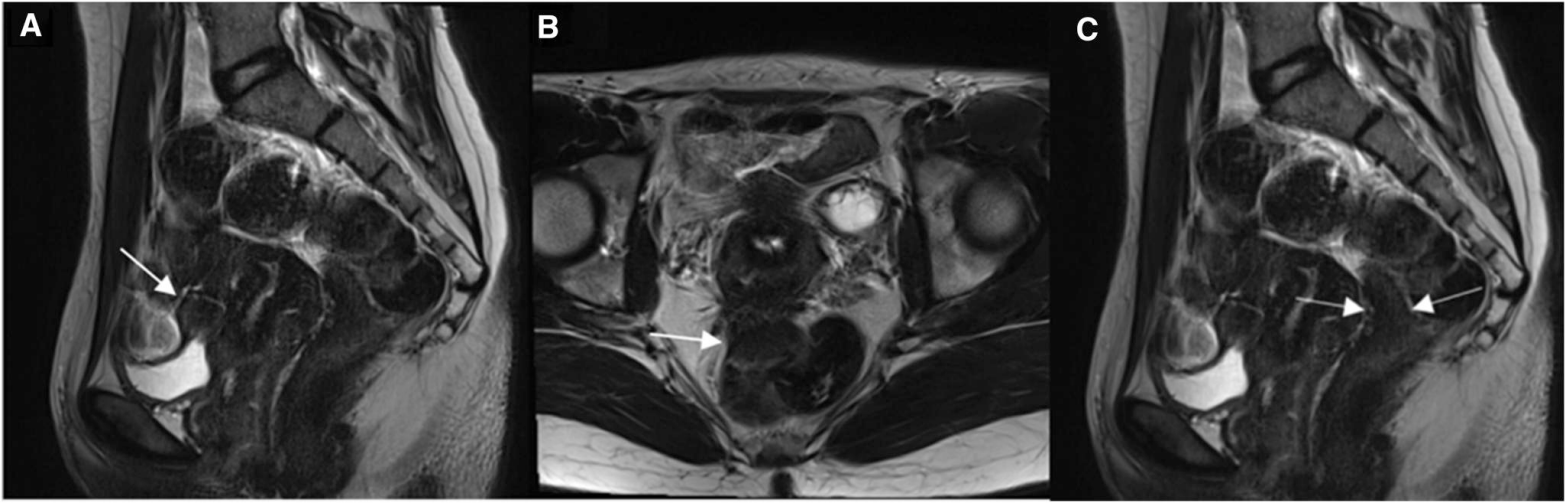
A 32-year-old female with history of DE (Case courtesy of Dr. Laura Fender). MRI pelvis showed (A) endometriotic nodule in the vesicouterine fold (arrow), (B and C) a large mushroom-shaped bowel DE nodule which is tethered to the posterior cervix (arrows).

MRI is a powerful non-invasive tool for the detection and characterization of endometriosis due to its detailed multiplanar visualization and ability to assess sonographic “blind spots” (Figure 10). It is a second line modality, usually used to stage endometriosis preoperatively or to monitor non-operative cases.^54^ Small field of view sequences provide comprehensive evaluation of the pelvis, while large field of view sequences allow assessment of extrapelvic involvement and complications such as bowel lesions and hydronephrosis.^20^ Endometriosis MRI protocols vary between institutions.^66^ Endometriosis MRI can be performed on both 1.5T or 3T scanners and the use of phased coil arrays placed over the pelvis and anti-peristaltic agents are highly recommended. At least partial urinary bladder distension is suggested. 3T scanners afford higher signal to noise ratio, higher spatial resolution for improved lesion detection and shorter acquisition times.^66^

The Society of Abdominal Radiology endometriosis disease-focused panel published a recommended MRI protocol for evaluation of pelvic endometriosis in 2020.^66^ The fundamentals of an Endometriosis MRI protocol include T2-weighted Spin Echo sequences without fat suppression ideally in 3 planes to provide anatomic detail and assessment of lesion morphology and signal. T2-weighted imaging provides the highest yield in the detection of DE.^67^ T1-weighted imaging (T1WI) with and without fat suppression in axial and sagittal planes is recommended to enable lesion characterization and detection of blood and fat-containing lesions.^54^ Fat saturated T1 post-intravenous contrast in sagittal and axial planes and diffusion weighted imaging (DWI) are also recommended.^66^^,^^68^ Whilst the diagnosis and staging of endometriosis can be performed without intravenous contrast and DWI, their use is recommended by several authors to assess for possible malignant transformation or infection.^42^^,^^66^ Additional sequences have been suggested such as cinematic T2-weighted sagittal motion sequences, which enable assessment of pelvic organ mobility.^69^ Susceptibility weighted imaging (SWI) has potential utility in improving detection of subtle extra-ovarian endometriosis and in the diagnosis of atypical endometriomas.^42^ Further optional techniques include use of vaginal gel to assess for vaginal DE and rectal gel, contrast or bowel preparation for the detection of bowel DE.^20^^,^^66^ The main limitations in the use of MRI in this setting include its high cost, limited accessibility, lengthy scan times, reporter variability and challenges with dynamic assessments.^70^

The accuracy of MRI for detection of endometriosis varies by location, with highest sensitivity, specificity and accuracy for rectosigmoid endometriosis of 100%, 93%, and 95%, respectively.^71^ There is excellent (91%-95%) diagnostic accuracy for detection of uterosacral ligament DE, rectovaginal septum DE and endometriomas, with 82% accuracy in the detection of ureteric involvement. MRI performs relatively poorly in the detection of superficial and small peritoneal deposits, with low accuracy and sensitivity.^71^ When visible, superficial endometriosis usually appears as small T1-hyperintense foci, or T2-hypointense thickening. Adhesions may complicate superficial deposits, manifesting as linear low T2 bands or tethering of viscera such as bowel.^54^^,^^66^

DE lesions are best perceived on T2-weighted imaging due to the contrast between low signal fibrosis and high signal fat. The appearance of DE lesions varies owing to the variable predominance of T2 low signal fibrosis and the presence of glandular components containing cystic (high T2 signal) or haemorrhagic (high T1 signal) foci.^42^^,^^54^ Morphologically, DE may be stellate and spiculated, irregular or smooth thickening, or nodular.^20^ DE lesions cause severe surrounding tissue distortion with obliteration of normal pelvic spaces (such as the Pouch of Douglas) or fixation of viscera.^20^ Visceral fixation infers more complex surgery of longer duration, with greater risk of complications.^54^

Uterosacral DE usually manifests as low T2 signal thickening and nodularity, possibly containing active haemorrhagic high T1 foci.

Bowel DE ranges in appearance depending on severity. Rectosigmoid colon DE is usually contiguous with DE involving the USL, torus uterinus, posterior vagina or retrocervical space and manifests as a T2 low signal tethering mass or mural thickening.^20^^,^^72^ DE invading the muscularis propria has mushroom-like morphology and the term “mushroom-cap sign” has been applied to the presence of the low T2 signal mass with high T2 signal overlying bowel mucosa.^20^^,^^73^ Small bowel lesions are more likely to be multifocal.

Urinary bladder DE may manifest as T2 low signal mural thickening or a discrete nodule projecting intraluminally.^20^^,^^74^ There may be associated obliteration of the vesicouterine space. When involved, the ureters are usually extrinsically compressed by a nodule with periureteric retractile adhesions.^20^^,^^54^ Endometriomas manifest as unilocular or multilocular ovarian or para-ovarian cysts containing markedly high T1, usually low T2 signal contents (known as T2 shading) owing to the presence of blood products of variable ages from repeated haemorrhage.^20^^,^^42^ The cysts may contain fluid-fluid levels. Less frequently, endometriomas may show high signal on T2. SWI can be used to demonstrate mural haemosiderin staining from repeated haemorrhage in these cases, improving diagnostic confidence. The walls of endometriomas are usually thick and of low T2 signal intensity due to a combination of fibrosis and haemosiderin.^20^^,^^74^ The Fallopian tubes are involved in 30% of cases and may show haematosalpinx, hydrosalpinx, intraluminal high T1 foci or mural thickening.^20^^,^^54^

Malignant transformation may occur both in endometriomas or in DE, most commonly in the rectovaginal space and rectosigmoid colon DE.^20^^,^^34^^,^^42^^,^^68^ Whilst endometriomas may show diffuse restricted diffusion, correlation with T2-weighted sequences and post-contrast imaging enables differentiation of malignancy from blood products. Intra-cystic chronic retractile clot shows very low T2 signal intensity, while malignant elements will show intermediate T2 signal intensity and more focal diffusion restriction with early arterial phase hyperenhancement.^20^^,^^34^^,^^42^^,^^68^ Subtracted post-contrast T1WI is recommended to assess for true enhancement in light of high background T1 signal intensity. Loss of previously demonstrated T2 shading in endometriomas is another feature associated with malignant transformation. Rapid growth of DE with increased T2 signal relative to fibrosis, in addition to diffusion restriction and early arterial enhancement should be considered suspicious for malignant transformation.^20^^,^^34^^,^^42^^,^^68^

The use of structured MRI reporting for endometriosis is recommended to ensure clear and clinically relevant communication of findings. VanBuren et al provide an example of a template report for Endometriosis MRI based on the anatomical compartmental approach (anterior, middle and lateral and posterior compartments and other sites). Since the emergence of the Enzian and #Enzian classification systems, dedicated MRI-based reporting systems have emerged which have shown high interobserver concordance and diagnostic accuracy for DE.^20^^,^^71^^,^^75^^,^^76^

#### Adenomyosis

**Figure 11.**
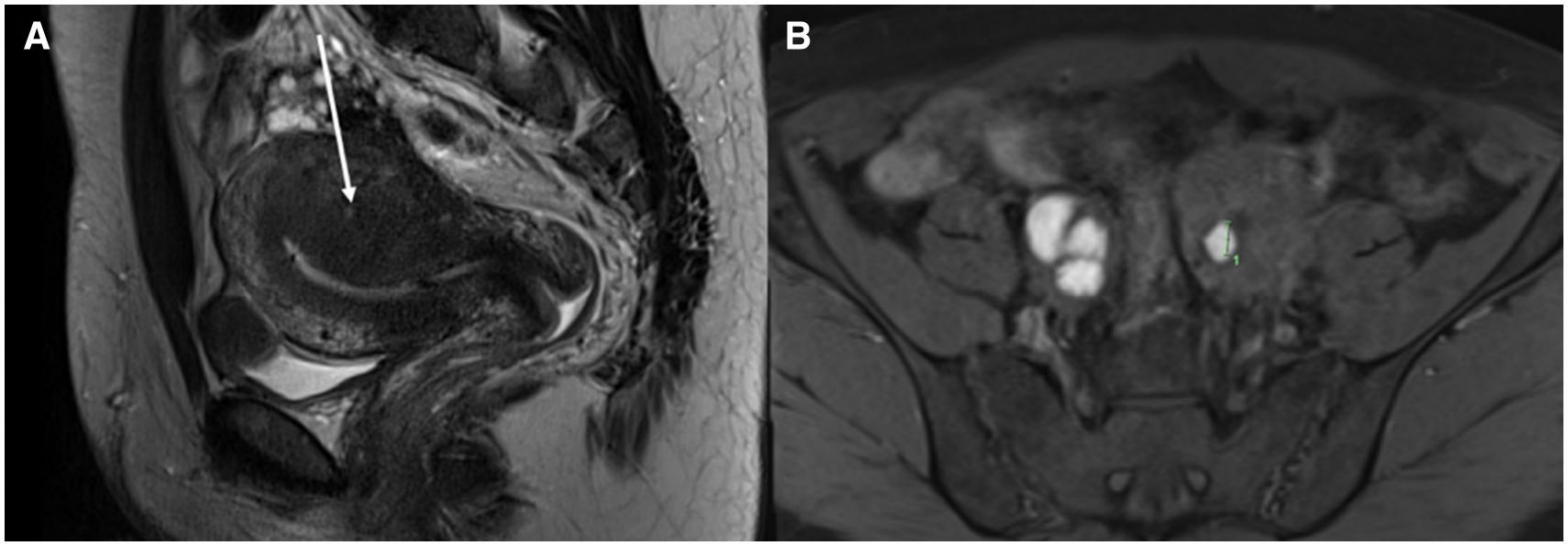
44-year-old female with a history of heavy menstrual bleeding. MRI demonstrated (A) a thickened junctional zone (arrow), in keeping with adenomyosis and (B) bilateral T1-hyperintense ovarian cysts consistent with endometriomas (caliper applied to a unilocular left endometrioma).

Adenomyosis manifests as thickening or irregularity of the junctional zone (>12 mm), subendometrial and myometrial cysts (which may be high T1 or high T2 signal depending on contents), asymmetric or global myometrial thickening and signal alteration and globular morphology of the uterus^20^^,^^77^ (Figure 11). Whilst there are no universally accepted classification systems, qualitative descriptors such as focal and diffuse adenomyosis and adenomyoma for more discrete mass-like lesions are commonly used. Similarly, localizing descriptors such as internal and external adenomyosis have been suggested to describe the inner and outer myometrium, respectively.^78^

### Other modalities

Although computed tomography (CT) may incidentally reveal endometriotic lesions during evaluation for other conditions, its routine use for endometriosis assessment is limited due to lower soft tissue contrast compared to other imaging modalities and radiation exposure.^79^ CT colonography shows good sensitivity of 82% for the detection of rectosigmoid DE, but specificity is poor at 67%.^71^ CT is generally reserved for problem solving in specific cases where other imaging techniques are inconclusive or in the assessment of endometriosis related complications.

To date, there is no radionuclide specific to ectopic endometrium-like tissue. FDG PET/CT shows low sensitivity and specificity in the detection of endometriosis lesions, with recognized variation in FDG avidity according to menstrual phase.^71^

Hysterosalpingography (HSG) and Hysterosalpingo contrast sonography (HyCoSy)/Hysterosalpingo foam sonography (HyFoSy) are commonly employed techniques for assessment of tubal patency in the evaluation of infertility, a complication of endometriosis. HSG uses contrast media injected into the uterine cavity, using fluoroscopic imaging to identify abnormalities in the uterus and fallopian tubes whilst HyCoSy/HyFoSy utilize US in conjunction with contrast agents to assess tubal patency.^80^

### Recent advancements and future directions

Elastography measures tissue elasticity and stiffness and its use is being explored in the assessment of endometriosis and adenomyosis. The systematic review by Brunelli et al^81^ showed that elastography had high sensitivity and specificity for DE and showed promise in the detection of adenomyosis and the differentiation of endometriomas from other ovarian masses.

Near-infrared fluorescence (NIRF) imaging is a real-time diagnostic tool for targeted intraoperative visualization of endometriosis. It uses ligands that bind to peptides, proteins, or molecules in endometriotic tissue, such as GnRH conjugated with infrared fluorophores, to generate fluorescence signals. This method enhances detection of small lesions and helps reduce residual disease after surgery.^82^

There is increasing interest in the role of artificial intelligence (AI) in aiding diagnosis of endometriosis and predicting surgical complexity and treatment success. However, there is limited high quality data currently available to inform algorithms and enable application of AI in this context.^83^

## Conclusion

Endometriosis and adenomyosis are complex conditions with high prevalence and diverse presentations that can lead to debilitating symptoms and significant complications. Radiologists, sonologists, and sonographers play pivotal roles within multidisciplinary teams in the diagnosis, treatment planning and follow-up of these conditions. Challenges persist in the variability of operator expertise, consistency of reporting, accuracy and accessibility. The lack of a universally accepted classification system further complicates diagnosis and management, though recent comprehensive proposals show promise. Encouraging steps are being taken to optimize current non-invasive diagnostic techniques and take advantage of emerging technologies to promote greater accuracy, personalized treatments and improved quality of life.
